# Interactions between the Bumblebee *Bombus pascuorum* and Red Clover (*Trifolium pratense*) Are Mediated by Plant Genetic Background

**DOI:** 10.1371/journal.pone.0161327

**Published:** 2016-08-23

**Authors:** Richard J. Sands, Jennifer K. Rowntree

**Affiliations:** Centre for the Genetics of Ecosystem Services, Faculty of Life Sciences, The University of Manchester, Michael Smith Building, Manchester, United Kingdom; Estacion Experimental de Zonas Áridas (CSIC), SPAIN

## Abstract

Wildflower mixes are often planted around field margins to provide forage for pollinators. Although seed for these mixtures is often wild-sourced, for species where agricultural cultivars are available, for example red clover (*Trifolium pratense*), cultivars can also be included. Previous evidence suggests that plant genetic background can have a strong influence on plant-arthropod interactions and therefore the provenance and genetic background of the plants included in wildflower mixes could impact plant-pollinator interactions. We tested the performance of five individual *T*. *pratense* cultivars against two commercially available wild-sourced *T*. *pratense* populations in terms of their ability to attract potential pollinator species (focusing on bumblebees) and their floral traits using greenhouse and garden experiments. The main bumblebee observed interacting with *T*. *pratense* was *Bombus pascuorum* and we found no difference in the absolute number of *B*. *pascuorum* visiting the cultivars or wild populations. However, we found variation among cultivars and between wild populations in their ability to attract bumblebees, which seems to be related to their relative investment in different floral traits. There was a positive relationship between biomass and number of inflorescences produced by the wild populations of *T*. *pratense*, which was not apparent for the cultivars. This suggests that artificial selection on the cultivars has changed the G-matrix of correlated traits. We show that agricultural cultivars of *T*. *pratense* can be as effective as wild populations at attracting pollinators such as bumblebees, but that the genetic background of both cultivars and wild populations can have a significant impact on the attractiveness of the plant to pollinators. We also show divergence in the correlated traits of *T*. *pratense* cultivars and wild populations that could lead to outbreeding depression if the plants interbreed.

## Introduction

Pollinators such as bumblebees provide important ecosystem services. However, factors such as agricultural intensification have caused a decline in pollinator abundance and diversity [1, 2] and, consequently, pollinators have become a research and conservation priority [3]. Many countries now have initiatives to encourage the provision of resources for pollinators, especially in areas of agricultural activity (e.g. UK Entry and Higher Level Environmental Stewardship Schemes [4, 5]). One method of providing these additional resources is by planting wildflower mixes around field margins [6]. Such environmental seed mixtures have been effective in providing foraging habitat for pollinators, for example bumblebees [6, 7], especially when plant species such as red clover (*Trifolium pratense* L.) are included [8], as the pollen of *T*. *pratense* is favoured by many bumblebee species [9].

Commercial ‘wildflower’ mixes may, however, differ from natural communities of wild flowers in the amount of resources available. As natural flower seed is an expensive commodity, species such as *T*. *pratense* may be represented in commercial mixes either as seed collected from natural populations or as a set of cultivars. Many agricultural cultivars of *T*. *pratense* have been bred as ley crops or forage where, for example, biomass (yield) and disease resistance traits have been preferentially selected for [10]. However, the selective breeding process may also alter floral phenology or morphology compared to wild counterparts [11]. Furthermore, each cultivar breeds true, meaning that there is generally reduced genetic variation within cultivars compared to natural populations. Reduced levels of genetic variation may lead to a reduction in population fitness [12], and within species genetic variation can have more profound, indirect effects on the wider ecosystem (e.g. [13]).

The environment that a species experiences can be influenced by the expressed genomes of interacting species; i.e. through the influence of extended phenotypes [13, 14]. Recent evidence suggests that floral abundance, flower visitor abundance and visitor taxonomic richness of multi genotype plant communities is greater than that of single genotype plant communities [15]. Additional studies have shown that the genetic identity of plants can determine the abundance of interacting arthropods both directly [16] and indirectly [17]. Therefore, understanding how genotypes or genetically distinct groups of a particular species influence interactions among species can be an important component of promoting plant-pollinator interactions. For example, the performance of wildflower mixes in terms of pollinator attraction could be improved by selecting plant genotypes that extend the flowering period, increase floral abundance or floral resources of the mixture as a whole.

We had two objectives with this study. The first objective was to test the efficacy of *T*. *pratense* cultivars in attracting arthropods, and in particular potential pollinators, compared to wild populations of the same plant species. The second objective was to determine if there was consistency in attraction ability among different cultivars or wild populations. In other words, we wanted to know if and how the genetic background of the plants influenced the plant-pollinator interactions.

## Materials and Methods

Red clover (*Trifolium pratense* L.) is a leguminous herb that is widely distributed though out Europe and had been cultivated for inclusion in pastures. Wild populations tend to be diploid (2n = 14) but many cultivated varieties are tetraploid [18]. The species is an obligate out-crosser and has a strong self-incompatibility system [19]. The circular inflorescences are comprised of numerous flowers [18], which provide nectar at the base of their corolla that is accessible to long tongued bees (e.g. *Bombus pascuorum*) or via nectar robbing [20]. Pollen of *T*. *pratense* is known to be collected by bumblebees [9].

### Garden experiment

#### Experimental design

Fifty 15 litre pots were planted with *T*. *pratense*. First, a layer of pea gravel was placed in the bottom of each pot for drainage and the pots were then filled with a mixture of low nutrient JI1 (John Innes no. 1; Keith Singleton, Cumbria, UK) compost and horticultural sand (33L: 25kg; Keith Singleton, Cumbria, UK). John Innes composts are a commonly used plant-growing medium with standardised ingredients (loam, peat, course sand) and fertilisers (see www.johninnes.info for more information). The sand and compost were combined using a concrete mixer and added into the pots. Twenty-five pots were planted with one of five cultivars and a further 25 were planted with plants from one of two wild populations. Seed from the five agricultural cultivars: Pavo (P); Rajah (R); Vesna (V); Amos (A) and Maro (M) were provided by Oliver Seeds (Lincolnshire, UK) and seed from two wild sourced populations (E) and (H) were supplied by Emorsgate (Kings Lynn, UK) and Herbiseed (Berkshire, UK) respectively. Two of the five agricultural varieties were diploid (cultivars P & R) and three were tetraploid (cultivars V, A and M). Seeds of each cultivar and population were sown in seed trays containing JI1 compost. Thirty-seven days after planting, seedlings were transplanted into the experimental pots. Each pot contained 20 plants of a single cultivar or population planted in a circular-grid pattern. Due to poor germination rates, there were not enough plants to create equal numbers of pots for each cultivar or population. Therefore, the final set up included five pots of cultivars A, P and V, three pots of cultivar M, 7 pots of cultivar R, 10 pots of population E and 15 pots of population H. Experimental pots were kept under glass for 23 days before being placed outside where pots were randomly positioned in a five row grid pattern on a plastic membrane at the experimental grounds of the University of Manchester (53°26’38.66”N, 2°12’49.88”W). Each row was 1 m apart and each pot was 0.5 m apart within a row. Pots were left for five weeks to acclimatise before surveying. At the end of the experiment in October 2013, above ground biomass was estimated for each pot by removing foliage 2 cm above the surface of the soil, drying at 80°C for 48 h and weighing.

#### Arthropod community assessment

The above-ground arthropod communities of the pots were surveyed once a calendar week for eight weeks between 20th^th^ May 2013 to 22^nd^ July 2013. Survey days varied from week to week and were selected as days when no rain was forecast. The number of inflorescences on each pot was recorded at the time of each survey. It was not possible to cover the flowers between surveys and, therefore, they were available to the local arthropod community over the whole time period of the experiment. Surveys were conduced three times on the chosen day with 5-hour intervals between each survey (7am, 12noon and 5pm). Times were chosen so that we would capture as much of the active period of the insects as possible. During a survey, the starting pot was chosen at random and then each pot was observed for 30 seconds in turn. Although short, this time frame allowed us to capture a snapshot of the arthropod community interacting with each pot while minimising the number of double counts of an individual on a particular pot (i.e. if an individual moved away and returned). All arthropods observed on the focal pot during the survey period were recorded to Order level, with any *Bombus spp*. identified to species level [21]. *Bombus terrestris* (L.) and *Bombus lucorum* (L.) were combined during observations due to difficulties differentiating workers of these species.

### Glasshouse experiment

#### Experimental design

Clover seeds of the five cultivars and two populations were germinated on wet towelling in the fridge and transplanted to seed trays after one week. Thirty-five days after planting, seedlings were transplanted to 140, 15cm diameter pots containing a mixture of JI1 and horticultural sand (33L: 25kg), prepared as previously, with four seedlings per pot. Twenty replicate pots for each cultivar (A, M, P, R, V) and wild population (E, H) were established. Pots were arranged randomly on benches, under glass at the experimental grounds of the University of Manchester (53°26’38.66”N, 2°12’49.88”W). The temperature in the glasshouse ranged between 7°C and 35°C during the growing period and plants were grown under natural light.

#### Floral trait assessment

As *T*. *pratense* produces inflorescences that are clusters of multiple small flowers, we first counted the number of flowers on one inflorescence per pot for each cultivar and population. Within the time frame of the experiment, not all pots produced inflorescences of suitable quality to enable the accurate counting of flowers. Therefore, final replicate numbers are as follows: P = 19; M&V = 18; A = 17; R = 10; H = 14; E = 8.

Pollen was collected from the same inflorescences used to determine the number of flowers. In order to obtain accurate pollen estimations and to prevent pollinators removing the pollen from the plants, voile fabric bags were placed over inflorescences for at least 48 h prior to sampling. The number of pollen grains per flower was estimated by removing three flowers from the covered inflorescence and placing them in a 1.5ml tube containing 200μl of 20% (v/v) ethanol (adapted from [22]). Flowers were later dissected to isolate the anthers using a dissecting microscope (Leica Wild M8) and fine forceps. The anthers from all three flowers were masticated using forceps to ensure release of pollen into the ethanol and mixed to homogenise the solution. Three pollen counts per sample were carried out using an improved neubauer haemocytometer. Ten microlitres of ethanol containing pollen was pipetted onto the haemocytometer and the number of pollen grains in each corner (0.04mm^2^) and centre square (0.04mm^2^) giving a total of five squares, were counted using a slide microscope (Leitz wetzlar, Dialux 20) (pollen touching the bottom and right side lines were included; the top and left lines were excluded). The number of pollen grains per flower was estimated using the following calculation:

$$\left(\frac{\frac{X}{5}\times \;25\;\times \;2000}{3}\right)\;\times \;f$$ (1)

Where *X* = the total number of pollen grains in five squares, divided by 5 gives the mean per square, multiplied by 25 gives the amount under the coverslip, and 2000 is the total dilution factor. This was then divided by the three flowers and multiplied by *f*, the total number of flowers on the inflorescence, to give an estimate of the total pollen per inflorescence.

### Statistical analysis

The numbers of arthropods per sampling period were summed per pot over the eight weeks to give an estimate of the total number of arthropods observed. Arthropod data was subsequently divided to give the abundance of *Bombus pascuorum* (Scopoli.), the most numerous *Bombus* species, and the abundance of Diptera, the most numerous order. The numbers of inflorescences counted over the eight-week observation period were summed per pot to give an estimate of the total number of inflorescences produced.

All data sets were first plotted to determine if there were any outliers or heteroscedasticity in the distribution of the data [23]. One data point was removed from the arthropod data set and two data points were removed from the *Bombus pascuorum* data set due to unusually high or low values. No data points were removed from either the Diptera or the inflorescence data sets. Each data set was then analysed first with a linear mixed effect model where treatment (cultivar or wild) was included as a fixed effect and plant type (specific cultivar or population) as a random effect. Then, the data were analysed with a generalised linear mixed effect model containing the same fixed and random effects, but with a poisson distribution, as all of the data collected was count data. The model fit was compared using AIC criteria, where the model with the lowest AIC value was considered a better fit [24]. Where the model with the poisson distribution was the best fit, this was then tested for overdispersion (following the approach suggested at http://glmm.wikidot.com/faq). Finally, a spatial component was added to the previous best-fit model with the inclusion as fixed effects of the x and y spatial coordinates of the pots and an interaction between the two. The fit of the spatial model was then compared, as previously, against the non-spatial model and the results of the best-fit model examined in more detail. A similar approach was undertaken for analysis of the number of flowers and amount of pollen per inflorescence, except that no model with a spatial component was tested and the pollen data was ultimately log transformed and analysed with a linear mixed model as this proved to be the best fit. The relationship between pot biomass and total number of inflorescences produced per pot in the garden experiment was also analysed using two linear regressions, one for the cultivars and the other for the wild populations.

All data analyses were undertaken in R version 3.2.3 [25]. Linear mixed effect models and generalised mixed effect models were analysed using the ‘lmer’ and ‘glmer’ programmes of the ‘lme4’ package respectively [26]. Significance values were assigned to fixed effects using the ‘Anova’ programme in the ‘car’ package with a type III model fit and Wald statistics [27]. The contribution of the random effects to the model fit were calculated using the ‘r.squaredGLMM’ programme in the MuMIn package [28]. Where the R^2^ value of the mixed effect model increased with inclusion of the random effects, this was taken as an indication that there may be significant differences among cultivars or populations. The random effects, with confidence intervals calculated using a random forest approach, were then plotted using the programmes ‘sjp.lmer’ or ‘sjp.glmer’ in the ‘sjPlot’ package [29]. Where confidence intervals did not overlap, cultivars or populations were deemed to be significantly different from each other. Graphics were produced using the package ‘ggplot2’ [30].

## Results

### Garden experiment

A total of 1035 arthropods from seven orders, including six *Bombus* species, were recorded over the eight weeks of observations (Table 1). Although the number of inflorescences per pot increased steadily between weeks 1–4, the majority were produced in the last four weeks of observations with high abundances remaining at week 8 when the observations ended.

**Table 1:** Total observed orders and species visiting *Trifolium pratense* between 20th May 2013 and 22nd July 2013.

Order			Number observed
Araneae			5
Coleoptera			97
Diptera			367
Hemiptera			74
Hymenoptera	(Non-*Bombus*)		68
	*Bombus*	*hortorum*	31
		*hypnorum*	1
		*lapidarius*	4
		*pascuorum*	351
		*terrestris & lucorum*	8
Lepidoptera			23
Neuroptera			6

The total abundance of arthropods was best explained by the linear mixed model without a spatial component. This confirmed a significant effect of plant treatment, whereby the wild populations of clover attracted a greater abundance of arthropods overall (χ^2^_1_ = 10.0, p = 0.002; S1 Fig). The random effects did not explain any variation in the model.

**Fig 1:**
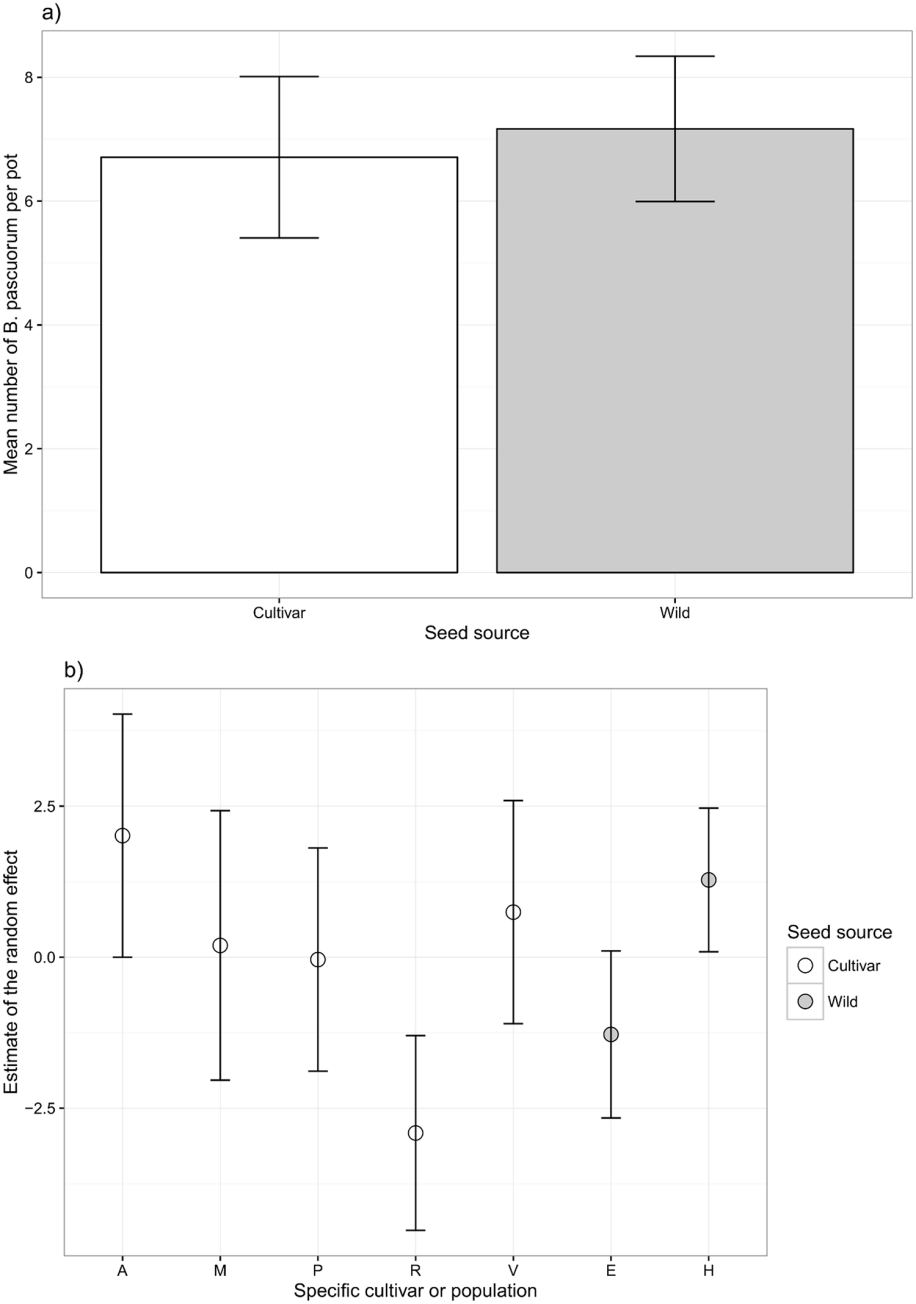
(a) Mean total abundance of *Bombus pascuorum* per pot for the cultivar (white) and wild (grey) treatments with 95% confidence intervals. (b) Estimate of the random effect for each specific cultivar (white) and wild population (grey) with 95% confidence intervals.

The total abundance of *B*. *pascuorum* was best explained by the linear mixed model without a spatial component. There was no significant difference between the abundance of *B*. *pascuorum* observed on the cultivars and the wild populations (χ^2^_1_ = 0.003, p = 0.96; Fig 1a). However, the random effects did increase the model fit (R^2^_without_ = 0.0001, R^2^_with_ = 0.31) and confidence intervals did not overlap between the cultivars R and A or V (Fig 1b), where the abundance of bees was lower on cultivar R. Confidence intervals were almost not overlapping between the wild populations E and H, where the abundance of bees was lower on population E (Fig 1b).

The total abundance data of Diptera species was best explained by the generalised linear mixed model with a poisson distribution, but without the spatial component. A test for overdispersion on the model suggested that data were not overdispersed (χ^2^_1_ = 53.71, p = 0.23). There was no significant difference in the abundance of Diptera observed on the cultivars or the wild populations (χ^2^_1_ = 1.18, p = 0.28; S2A Fig). As for *B*. *pascuroum*, the random effects increased the fit of the model (R^2^_without_ = 0.059, R^2^_with_ = 0.26) and confidence intervals did not overlap between wild populations E and H (S2B Fig), where the abundance of Diptera was higher on population E.

**Fig 2:**
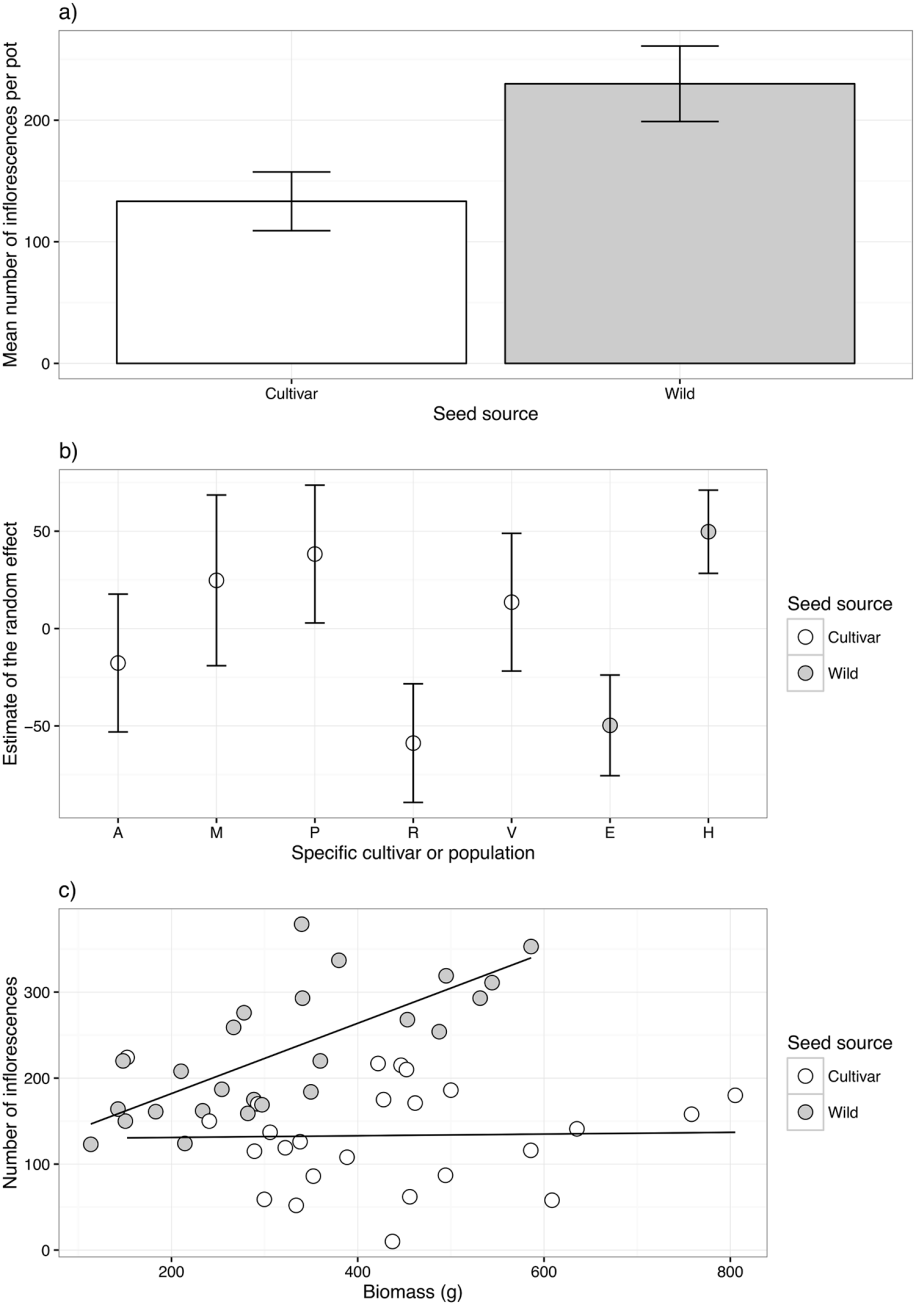
(a) Mean total abundance of inflorescences produced per pot for the cultivar (white) and wild (grey) treatments with 95% confidence intervals. (b) Estimate of the random effect for each specific cultivar (white) and wild population (grey) with 95% confidence intervals. (c) The relationship between biomass (g) and number of inflorescences produced within each pot. Cultivars are shown in white and wild populations in grey. Lines are fitted linear regressions. There is a significant relationship between biomass and inflorescence number for the wild populations (p = 2.4 x 10^−5^), but not for the cultivars (p = 0.90).

The total abundance of inflorescences was best explained by the linear mixed model with the inclusion of a spatial component. There was a marginally significant difference between cultivars and wild populations with the wild populations producing more flowers (χ^2^_1_ = 3.6, p = 0.058; Fig 2a). There were no significant effects of the pot x (χ^2^_1_ = 0.73, p = 0.39) and y (χ^2^_1_ = 1.6, p = 0.21) coordinates and no interaction between them (χ ^2^_1_ = 0.061, p = 0.80). The random effects increased the fit of the model (R^2^_without_ = 0.36, R^2^_with_ = 0.73) and confidence intervals did not overlap between cultivar R and V, P & M with R producing fewer flowers than the others (Fig 2b). Confidence intervals also did not overlap between populations E and H with H producing more flowers than E (Fig 2b).

Linear regression showed there to be no relationship between pot biomass and the number of inflorescences produced by the cultivars (R^2^_adj_ = -0.043, t = 0.13, p = 0.90). However, for the wild populations, there was a significant positive relationship between biomass and inflorescence number (R^2^_adj_ = 0.53, p = 2.4 x 10^−5^; Fig 2c)

### Glasshouse experiment

**Fig 3:**
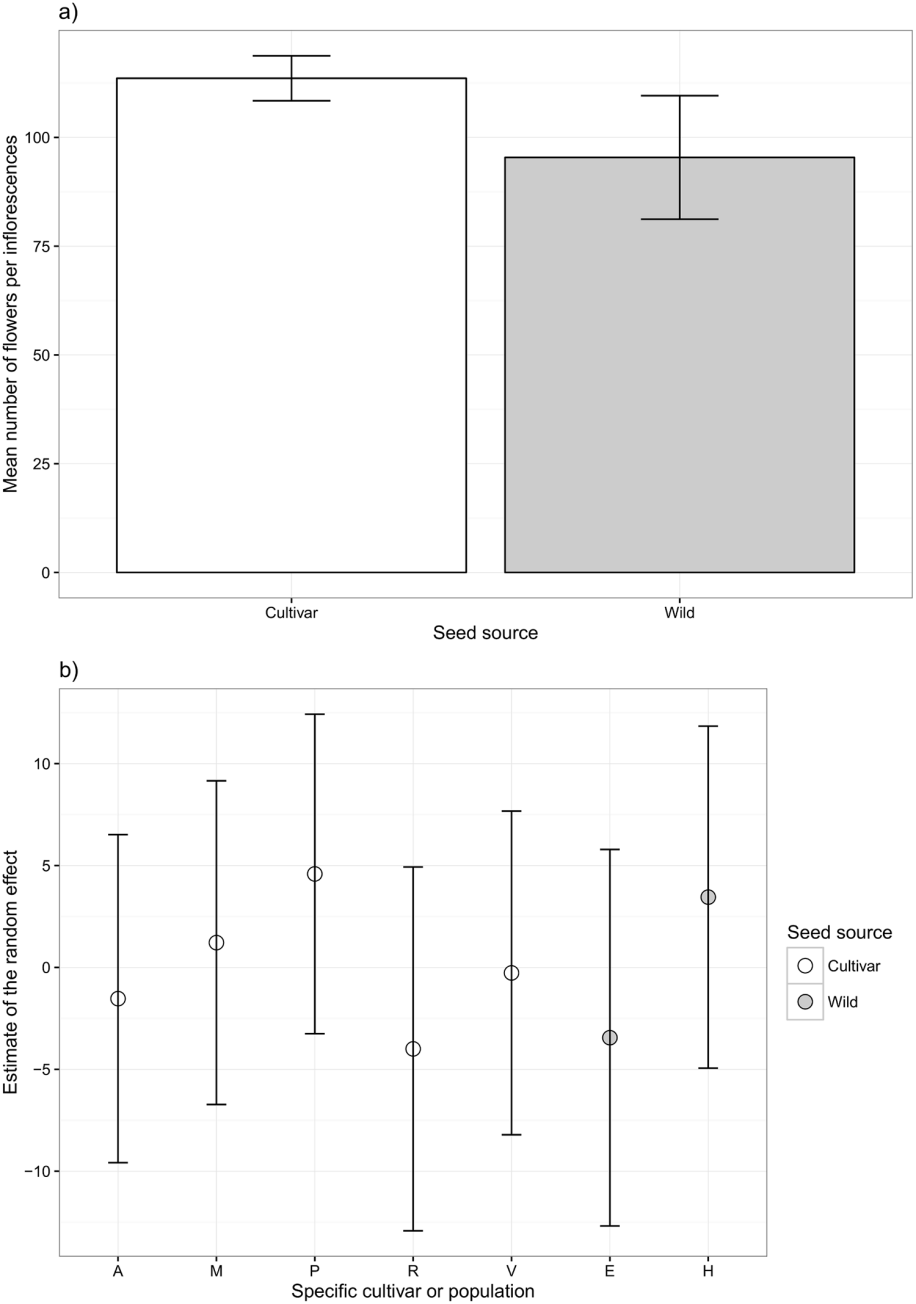
(a) Mean number of flowers per inflorescence for the cultivar (white) and wild (grey) treatments with 95% confidence intervals. (b) Estimate of the random effect for each specific cultivar (white) and wild population (grey) with 95% confidence intervals.

The number of flowers per inflorescence was best explained by the linear mixed model. This demonstrated a significant effect of plant treatment, with the cultivars producing larger inflorescences comprised of more individual flowers (χ^2^_1_ = 5.9, p = 0.015; Fig 3a). The random effects increased the fit of the model slightly (R^2^_without_ = 0.08, R^2^_with_ = 0.12), but confidence intervals among the cultivars and between the populations all overlapped (Fig 3b).

**Fig 4:**
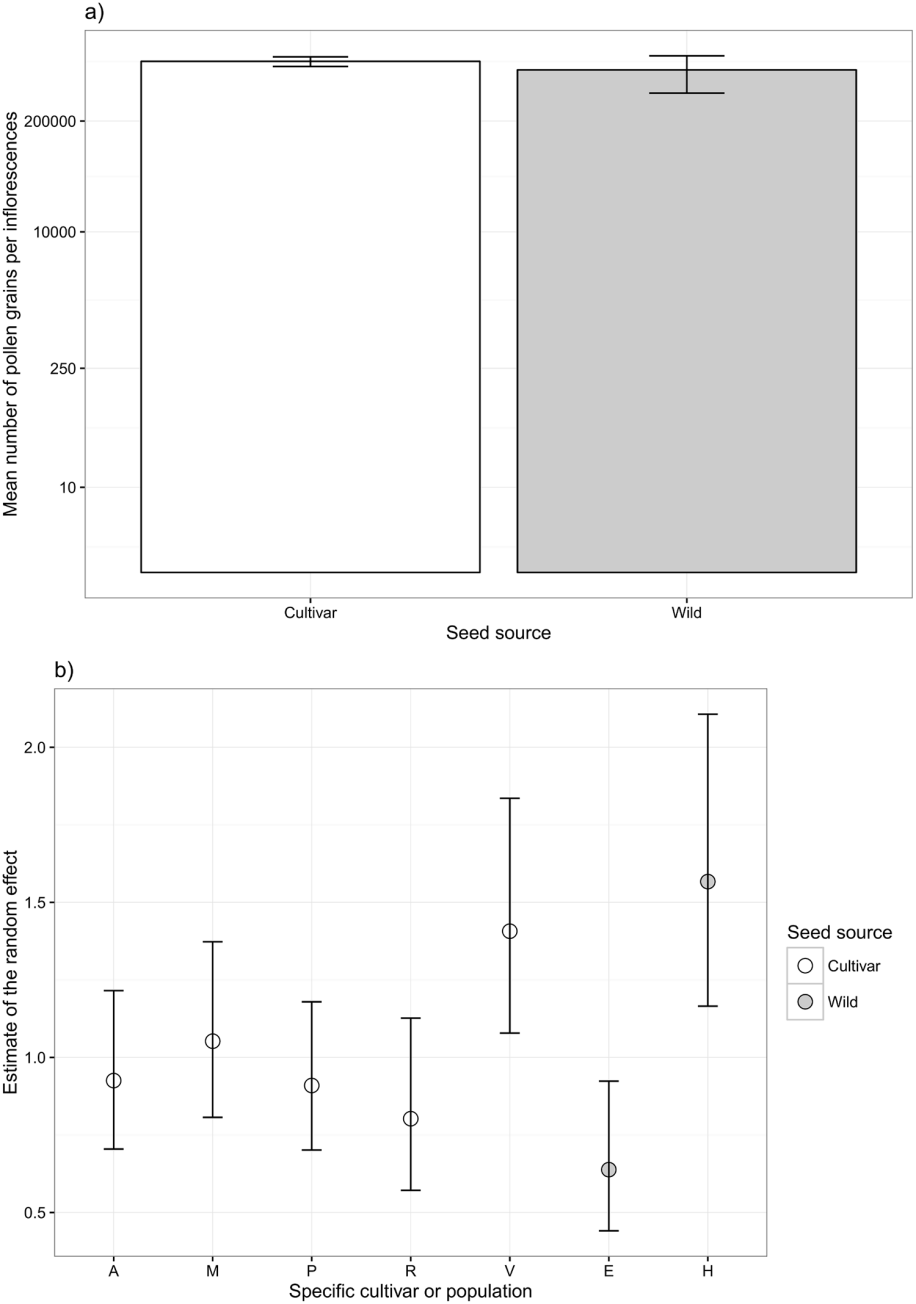
(a) Mean amount of pollen produced per inflorescence for the cultivar (white) and wild (grey) treatments with 95% confidence intervals. Note the logarithmic scale on the y-axis. (b) Estimate of the random effect for each specific cultivar (white) and wild population (grey) with 95% confidence intervals.

The amount of pollen per inflorescence was best explained by the linear mixed model after the data had been natural log transformed. There was no significant difference between the cultivars and the wild populations in the amount of pollen produced (χ^2^_1_ = 2.5, p = 0.11; Fig 4a). However, inclusion of the random effects increased the fit of the model (R^2^_without_ = 0.09, R^2^_with_ = 0.33) and confidence intervals between populations E and H did not overlap, with population H producing more pollen. Confidence intervals between the cultivars R and V were almost separated from each other, with cultivar V producing more pollen (Fig 4b).

## Discussion

Overall, we found that the wild populations of clover attracted a greater number of arthropods than the cultivars over the study period. However, this effect disappeared when we focused on the two main pollinating groups (*B*. *pascuorum* and Diptera). Instead, the abundance of bumblebees on the clover depended on the specific cultivar or population examined. For the Diptera, no differences were observed among cultivars, but the two wild populations attracted very different numbers of individuals. For the wild populations, the patterns in attraction of bumblebees and flies were in opposing directions, with population H attracting more bumblebees and population E more Diptera.

We attempted to reduce the probability of counting the same individual arthropod per pot multiple times by using a short observation period. However, the same individual could have been counted again on a different pot and we tried to account for this in a number of ways. First, pots were randomly arranged in a grid pattern so that no treatments were clumped together and the starting point of each observation period was randomly selected to reduce any positional bias in the data. Second, by sampling multiple times per day and over a number of weeks we hoped to reduce the chance of sampling the same individuals, while maximising our chance of sampling the whole arthropod community. Finally, as we did not find that the addition of the pot spatial coordinates improved the fit of any of the arthropod models and we saw no strong trends in the model residuals, we concluded that the position of the pot was not as important in determining the community composition as the specific plant treatments tested.

### Differences between cultivars and wild populations

The increase in arthropod abundance observed on the wild populations compared to the cultivars corresponds to the differences in the number of inflorescences produced by the plants as overall the cultivars produced fewer flowers. Previous research [31] has shown a positive relationship between the abundance of bumblebees observed and the number of inflorescences produced by a plant, and we would have expected the *B*. *pascuorum* abundance data to follow this same pattern. However, intraspecific variation, either among the cultivars or the wild populations, was more important in explaining the patterns in *B*. *pascuorum* abundance observed than differences between cultivars and wild populations *per se*. Our results suggest, therefore, that in general cultivars of red clover are as effective in attracting *Bombus pascuorum* as wild populations, and that there is no reason not to include them in pollinator wildflower mixes. Previous research has demonstrated that while cultivars are good at attracting pollinators when used in wildflower mixes on field margins, they don’t provide a sustained floral resource throughout the season [32]. As there was still a high abundance of inflorescences during the final week of our observations, we are unable to offer further insight into this finding.

Our results also suggest that bumblebee preferences are not only determined by inflorescence density. Heinrich [33] suggested that there is pressure on bumblebees to select the most rewarding flowers, in terms of calorific gains, from within a foraging area. Therefore, foraging efficiency would be improved if bees foraged over a reduced area on more rewarding flowers. In our glasshouse study, we found that the cultivars produced larger inflorescences with a greater number of individual flowers than the wild populations. As each individual flower on a clover inflorescence is a potential source of nectar, the larger number of flowers on the cultivars could indicate that they contain a higher volume of nectar, and are therefore more energetically rewarding to the bees. However, previous studies have not necessarily found this relationship to be true [34]. Unfortunately, we were unable to collect nectar in sufficient volume and in a reproducible manner during this study, so were unable to test this hypothesis. This would be an obvious important question to answer in future studies.

When we plot the number of inflorescences produced against biomass for each pot, we find a significant positive relationship for the wild populations but no significant relationship for the cultivars. This suggests, firstly, that there is a genetic correlation between inflorescence number and biomass for the wild populations [35] and, secondly, that this has been broken following artificial selection of the cultivars. As clover cultivars have generally been developed for forage, where floral traits are not the primary concern [10], it is unlikely that a disruption of the biomass-inflorescence relationship has been directly selected for. However, the change in a genetic correlation between inflorescence number and biomass in the cultivars means that the cultivars and wild populations may respond differently from each other when subject to additional selection pressures [36, 37]. Thus, cross-pollination between cultivars and wild populations of red clover could lead to outbreeding depression [38]. Therefore, while there is no reason not to include red clover cultivars in wildflower mixes if the focus is to attract bumblebees, if we also consider the longer-term viability of the plant population, some caution should be used when selecting cultivars for inclusion.

### Impact of plant genetic background

It is also clear from our results that at the cultivar or population level, careful choice of seed for environmental mixtures needs to be made, as some populations and cultivars are considerably better at attracting bumblebees and Diptera species than others. The variation observed in the abundance of *B*. *pascuorum* among the cultivars and populations shows similar patterns to the number of inflorescences produced and the amount of pollen per inflorescence. As well as nectar, pollen is an important resource for bees, as it provides protein and essential amino acids that are necessary for the healthy development of larvae [33], and Fabaceae pollen is particularly valued by *Bombus* species [9]. Population H produced a high abundance of inflorescences containing substantial amounts of pollen, which corresponds with a greater attractiveness of H to the bees compared to population E. Cultivar R had comparatively low inflorescence production, while cultivar V had inflorescences well provisioned with pollen. This combination of traits corresponds to the relative attractiveness of these cultivars to *B*. *pascuorum*, but does not explain why the highest number of bees were observed interacting with cultivar A.

Broadly, we found that among cultivars and populations the trends observed in the abundance of Diptera opposed those of *B*. *pascuorum*. Higher abundance of Diptera was observed on population E compared to H, while cultivar V did not appear to be attractive to the flies. Although an important pollinator group [39], the Dipteran visits in this case did not seem to reflect interactions indicative of pollination, as the majority of Diptera appeared to be simply resting (R Sands, personal observation). This behaviour is characteristic of some Diptera, which thermoregulate by basking [40] and the greater number of interactions between Diptera and the wild population E may reflect favourable basking conditions [41], as this population had spreading growth and low stature plants.

Much of the variation observed within cultivars, both in floral traits measured and in the ability to attract bees, is due to a single cultivar R. This cultivar is one of the diploid cultivars that we used and the oldest developed cultivar tested (Rod Bonshor, personal communication). Some of the observed differences in cultivar R may be due to its ploidy level, as polyploid plants are generally larger than their diploid counterparts, both in terms of biomass production and their floral structures [42]. However, similar patterns were not observed for cultivar P, the other diploid cultivar used in this study.

The fact that we see differences among cultivars or populations of clover in their ability to attract bumblebees and flies when grown together in a common garden experiment, strongly suggests that underlying genetic variation within the plants influences plant-pollinator attractions [43], although maternal effects could also explain some of the variation observed [44]. Here, we took agricultural cultivars to be genetically distinct groups as they have all undergone selection and been bred for a series of reproducible traits [42], and different populations, especially if geographically distant, are likely to be genetically differentiated [45]. Previous studies using similar common garden experiments have shown that genetic variation among genotypes can have a considerable effect on the outcome of plant-arthropod interactions influencing the number of pollinators [15], herbivores [16] and the general arthropod community [46]. Our results, therefore, add to an increasing body of evidence that plant genetic diversity is an important component of plant-arthropod interactions. More specifically, they suggest that while both cultivars and wild populations of red clover are effective in attracting bumblebees to an area, genetic differences both among cultivars and between populations of plants can determine the efficacy of this effect.

## Supporting Information

S1 Data Raw data files from the garden experiment. (CSV)

S2 Data Raw data files from the greenhouse experiment. (CSV)

S1 Fig Mean total abundance of arthropods per pot for the cultivar (white) and wild (grey) treatments with 95% confidence intervals. (TIFF)

S2 Fig Mean total abundance of Diptera per pot for the cultivar (white) and wild (grey) treatments with 95% confidence intervals. Note the logarithmic scale on the y-axis. (b) Estimate of the random effect for each specific cultivar (white) and wild population (grey) with 95% confidence intervals.
(TIFF)
