# Supplementary figures and images for: Interactions between the Bumblebee Bombus pascuorum and Red Clover (Trifolium pratense) Are Mediated by Plant Genetic Background
Source: PLoS One. 2016 Aug 23;11(8):e0161327. doi: 10.1371/journal.pone.0161327 (PMC4995044; doi:10.1371/journal.pone.0161327)

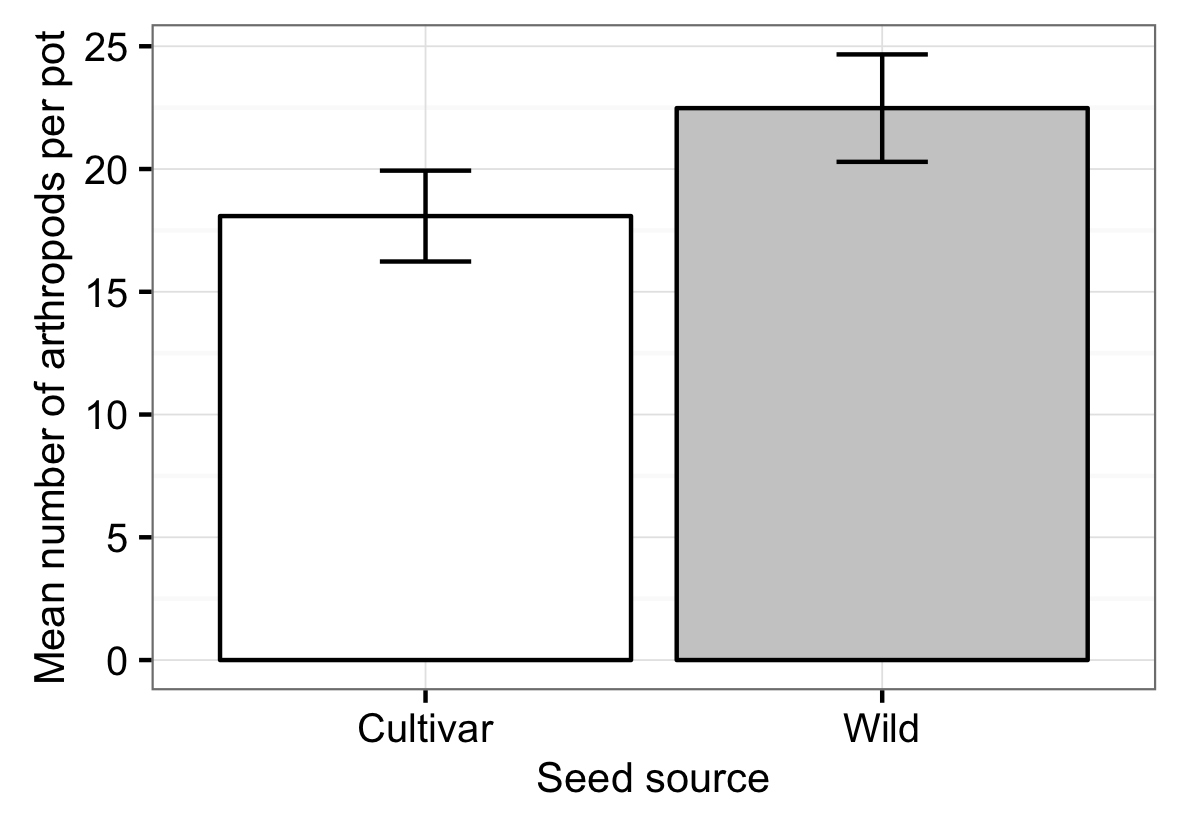

Supplement: S1 Fig — (TIFF) [file pone.0161327.s003.tiff]

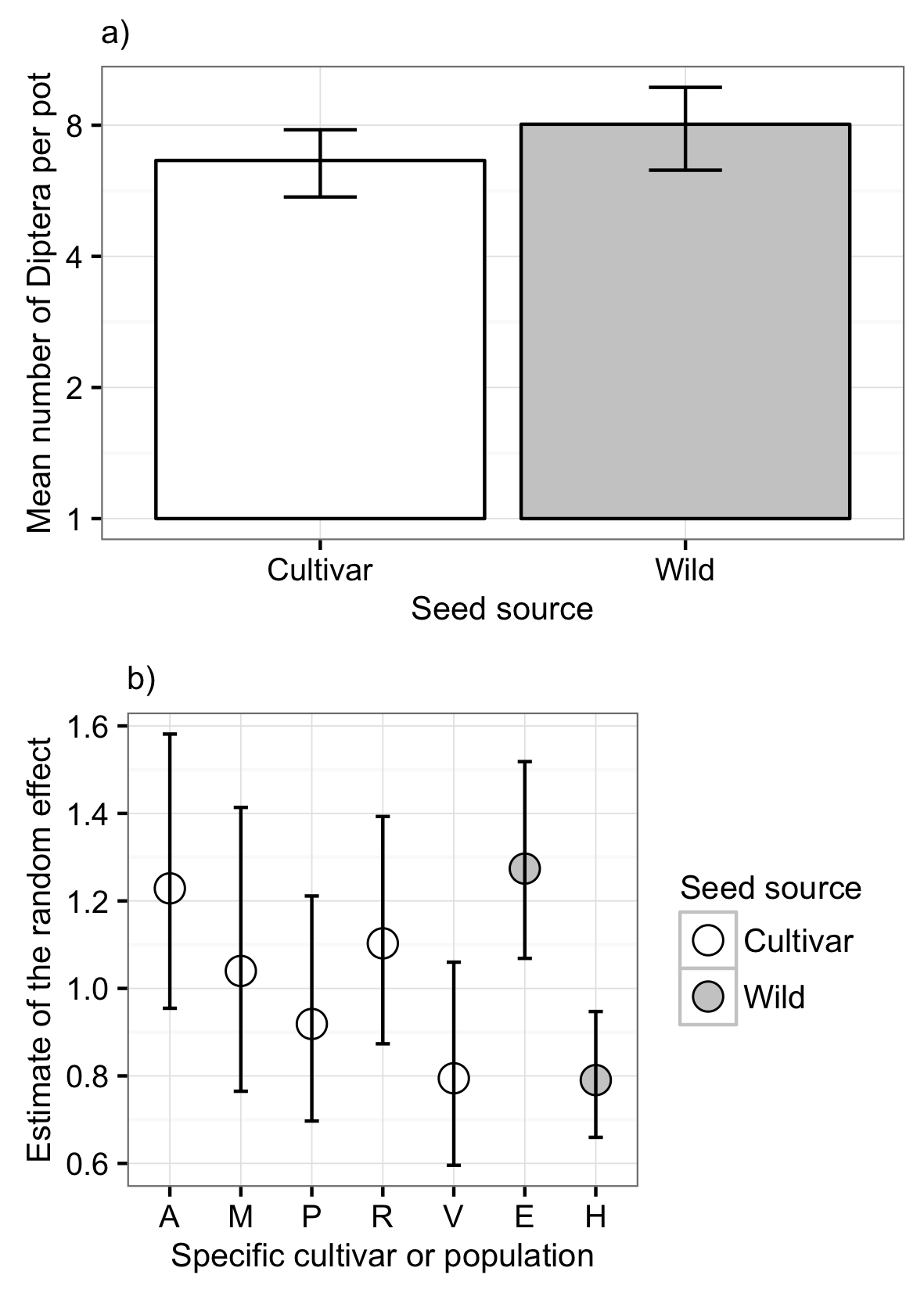

Supplement: S2 Fig — Note the logarithmic scale on the y-axis. (b) Estimate of the random effect for each specific cultivar (white) and wild population (grey) with 95% confidence intervals. (TIFF) [file pone.0161327.s004.tiff]
